# Lightweight, highly tough and durable YBa_2_Cu_3_O_7__–x_ superconductor

**DOI:** 10.1093/nsr/nwad030

**Published:** 2023-02-10

**Authors:** Baoqiang Zhang, Xingyi Zhang, You-He Zhou

**Affiliations:** Key Laboratory of Mechanics on Disaster and Environment in Western China attached to the Ministry of Education of China, Lanzhou University, Lanzhou 730000, China; Department of Mechanics and Engineering Sciences, College of Civil Engineering and Mechanics, Lanzhou University, Lanzhou 730000, China; Key Laboratory of Mechanics on Disaster and Environment in Western China attached to the Ministry of Education of China, Lanzhou University, Lanzhou 730000, China; Department of Mechanics and Engineering Sciences, College of Civil Engineering and Mechanics, Lanzhou University, Lanzhou 730000, China; Key Laboratory of Mechanics on Disaster and Environment in Western China attached to the Ministry of Education of China, Lanzhou University, Lanzhou 730000, China; Department of Mechanics and Engineering Sciences, College of Civil Engineering and Mechanics, Lanzhou University, Lanzhou 730000, China

**Keywords:** YBCO superconductor, lightweight, toughness, durability, interlocking dual network

## Abstract

The inherent brittleness and low sustainability of YBa_2_Cu_3_O_7__–x_ (YBCO) bulk superconductor seriously impede its wide applications. It is a great challenge to achieve toughening of this material and maintain its invariable superconductivity at the same time. Here, we fabricate bulk YBCO composite superconductor with a density of 2.15 g cm^−3^, which consists of interlocking dual network construction and shows high toughness and durability. The results show that its unit normalized fracture energy at 77 K reaches 638.6 kN m^−2^, which is ∼14.8 times that of YBCO bulk prepared by the top-seeded melt textured growth (TSMTG) method. Its critical current shows no degradation during the toughening process. Moreover, after 10 000 cycles, the sample does not fracture with the decay of critical current at 4 K of 14.6% whereas the TSMTG sample fractures only after 25 cycles.

## Teaser

A novel interlocking dual network construction YBCO composite superconductor were prepared and reported, which represent extraordinary properties including lightweight, high toughness, and durability to solve brittleness of ceramics.

## INTRODUCTION

In 1986, a new type of ceramic cuprate material YBa_2_Cu_3_O_7__–x_ (YBCO) was discovered with a transition temperature exceeding the temperature of liquid nitrogen [1]. This discovery greatly encouraged the exploration of new types of superconductors in the liquid nitrogen temperature region since the cost of superconductivity achievements was significantly reduced [2]. Despite the obstacle of the intrinsic brittleness of YBCO, its excellent superconducting properties attract researchers to solve the limitation of insufficient toughness without stopping [3–5]. The second-generation high-temperature superconducting tape has been fabricated by depositing YBCO on an alloy substrate with good mechanical properties, which can realize large-scale industrial production and has become the main approach of high-temperature superconducting applications at present [6,7]. As another main product form of YBCO material, bulk material has important applications [8–10] in superconducting maglevs, bearings, magnetic shielding, switches, etc. The conceptual prototypes of these devices were established many years ago, but they have not been applied in large-scale engineering. The main reason is that the processing and durability problem of YBCO superconducting bulk has not been effectively solved. Generally, the bulk YBCO superconductors were produced by the ‘top-down’ manufacturing processes [11], such as top-seed melting texture, melting texture growth and powder melting, etc. Although the weak connections at the grain boundary have been gradually overcome and the critical current density has increased, the mechanical properties are very low. The fracture toughness at 77 K is <3 MPa m^1/2^ in both the *ab* plane and along the *c*-axis direction [5]. Moreover, there are also many random microcracks caused by thermal stress generated during the sintering and cooling process induced by multiphase coexistence [12]. Combined with the cold–hot cycle and electromagnetic force in the application process, it is easy to accelerate microcrack propagation and cause the superconducting materials to fracture [13,14]. Although a great deal of work has been done, such as doping Ag, Cu, Au and other micro-nano metal particles [15,16], superconducting bulks wrapped with stainless steel and Ni–Cr alloy [17,18], epoxy impregnated bulks [5,19,20], carbon fiber, SiC fiber and glass fiber reinforcement [21–24], the toughening of YBCO bulk with high durability is still rare because these approaches all rely on external passive enhancement and uncontrollable preparation methods. Many biological materials such as mother of pearl [25,26], fintail mantis shrimp [27,28] and iron beetle [29] have an ordered multilevel microstructure, polymer chains and regular self-locking effect at the interface, showing both high strength and high toughness in synthetic or natural form [30]. Inspired by these results, thence, based on direct ink writing (DIW) 3D printing technology [31] and low-temperature cold casting, a lamellar skeleton structure of YBCO is formed. After the impregnation of epoxy resin, a YBCO composite bulk with tablet interlocking network microstructure is fabricated. The state-of-the-art YBCO superconductor achieves some unprecedented properties including being lightweight with high toughness and durability. Notably, the superconductivity shows no degradation during this toughening process. Our study demonstrates a credible route to transform a brittle functional ceramic into a lightweight, high toughness and durability composite material without function degradation.

## RESULTS AND DISCUSSION

### Processing strategy

**Figure 1.**
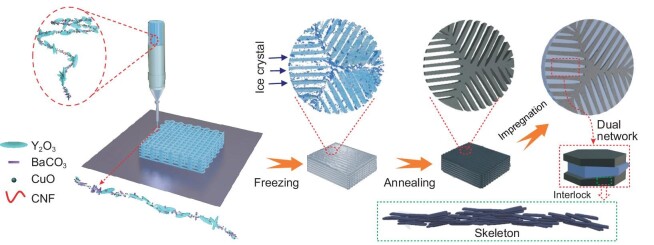
Schematic of fabrication of EPIP by 3D printing followed by freeze-drying and epoxy impregnation.

Figure 1 shows a schematic of our ‘bottom-up’ approach to impregnate 3D-printing YBCO (denoted as YBCO-3D) with epoxy resin (denoted as EP) to generate composite superconductors (denoted as EPIP). The fabrication procedure is described in detail in the Supplementary materials (SM, S1). In brief, Y_2_O_3_, BaCO_3_ and CuO powders were ball-milled to obtain well-dispersed powder (Supplementary Fig. S1). Subsequently, by roller milling and grinding, the paste composed of YBCO precursor particles, cellulose and soybean oil is obtained. Supplementary Fig. S2A–I shows the good rheology and plastic properties of the extrusion paste, including the printed 3D structures. To achieve an ordered microstructure, the printed wet samples are prepared by freezing-induced assembly, which promotes intimate contact and ordered orientation among the precursor particles under the ice crystals growing. The scanning electron microscope (SEM) images of the extruded round rod paste drawn in Supplementary Fig. S3 show the alignment of the Y_2_O_3_ and BaCO_3_ along the fiber direction before the sintering. Then, the freeze-drying green body was annealed and oxygenated to obtain superconductivity (this 3D printing process is schematized in Supplementary Fig. S4). Finally, EP was impregnated into the 3D YBCO porous skeleton to form EPIP with assisted heating and vacuuming (S2 in SM and Supplementary Fig. S5).

### The structural and crystal characterization of EPIP

**Figure 2.**
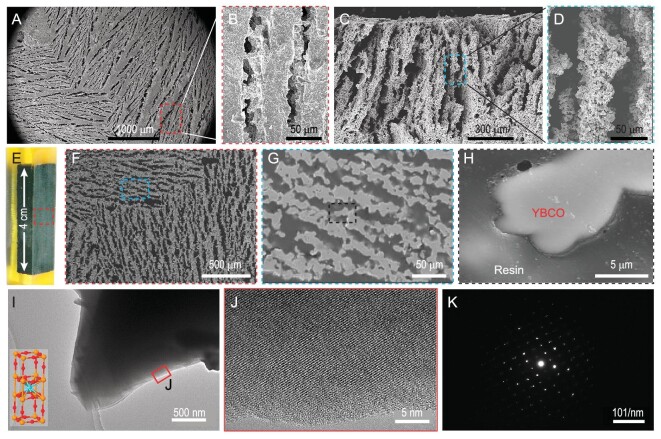
The structural and crystal characterization of EPIP. (A) and (B) SEM images of the surface section of YBCO-3D superconductor bulk after freeze-drying and annealing. (C) and (D) SEM images of the cross section of YBCO-3D superconductor bulk after freeze-drying and annealing. (E) EPIP with a density of ∼2.15 g cm^−3^. (F)–(H) SEM images of the cross section (perpendicular to the ice crystal growth direction). (I) Transmission electron microscope (TEM) image of the YBCO component of EPIP (the molecular ball-and-stick model of YBCO in bottom-left inset). (J) High-resolution TEM image. (K) The selected area electron diffraction (SAED) pattern corresponding to (J) clearly show the good crystallinity of the YBCO component.

As shown in the SEM images in Fig. 2A–D, the sintered 3D-printed bulk has a staggered porous skeleton structure. The orderly oriented alignment of the printed green body was formed after freeze-drying due to the growth of ice crystals and there are many bridging points and apophysis between the skeleton layers. Figure 2E shows the composite material morphology formed by the YBCO-3D lightweight bulk with a density of 2.15 g cm^−3^ after epoxy impregnation, which is ∼1/3 of the top-seeded melt textured growth (TSMTG) sample (Supplementary Table S1). The discussion about its density and cumulative pore volume by mercury intrusion test as well as the thermal analysis carried out by using thermogravimetric (TGA) and differential scanning calorimetry (DSC) are described in detail in Supplementary Figs S6–S8 and Supplementary Table S2, which indicate that the composite material is dense and stable. As can be seen from Fig. 2F and G, EP and YBCO-3D sheet can be well combined and there are no voids, bubbles, etc. in the whole area. From Fig. 2H, the interface of the epoxy resin and YBCO crystalline grain was robustly integrated without separation, peeling, gaps and cracks. There are microscale protrusions on each layer of YBCO-3D, which increase the interfacial resistance between EP and YBCO, forming a self-locking interface [32]. This structural characteristic can bring about large deformation capacity without structural instability, which is similar to the lamellar structure of mother of pearl aragonite [25,26]. The 3D microstructure of the EPIP bulk was reconstructed by using slice images after CT scanning (Supplementary Fig. S9). One can see that the ice crystals that extended from different directions of the 3D space led to the formation of a short-range ordered staggered YBCO skeleton structure (Movie 1). We now discuss the crystal characterization of EPIP. As shown in Fig. 2I–K, we find a typical layered perovskite structure (the molecular ball-and-stick model (Fig. 2I, bottom-left inset)) and the good monocrystalline nature of the YBCO component (Fig. 2J and K). The results indicate that the crystal structure in the composite has not been influenced by epoxy impregnation. The elemental mapping images of Y, Ba, Cu, O and C (Supplementary Fig. S10) and the X-ray diffraction (XRD) analysis (Supplementary Fig. S11) have confirmed a single superconducting Y-123 phase, which rules out the effect of the resin impregnation process. The crystal structures of the EPIP samples based on electron backscattered diffraction (EBSD) technology are displayed in Supplementary Fig. S12. It can be seen that EPIP has fine grain structure in the range of 5–20 microns per strip, according to the pole figure distribution drawn in Supplementary Fig. S13A and the distribution of the Kernel Average Misorientation map (Supplementary Fig. S13B and C), in which the maximum value of the local misorientation (LocMis) is 0.4, indicating a lower stress concentration in EPIP.

### Mechanical performances of EPIP

**Figure 3.**
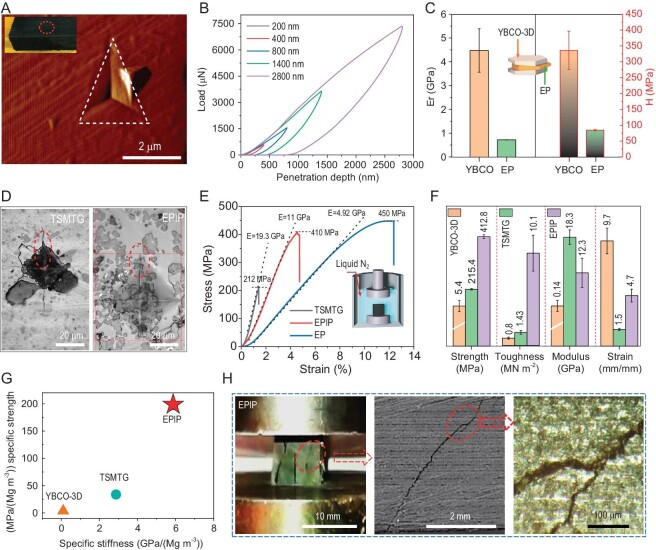
Mechanical properties of EPIP. (A) The residual indent image of the YBCO scaffold of bulk tested by using Atomic Force Microscopy (AFM). (B) The load–displacement curve of the YBCO scaffold of EPIP. (C) Microscopic elastic modulus and hardness YBCO scaffold and epoxy resin of EPIP. (D) Optical micrograph showing a Vickers indentation introduced on a polished section of TSMTG and EPIP. (E) Axial compressive stress–strain curves of typical TSMTG, EPIP and EP under 77 K. (F) Comparison of yield strength, toughness, elastic modulus and fracture strain for YBCO-3D, TSMTG and EPIP at 77 K. (G) Comparison of specific strength and specific stiffness of YBCO-3D, TSMTG and EPIP materials at 77 K. (H) Fracture surface of EPIP, crack deflection and branching at room temperature.

The microscopic mechanical properties of EPIP were studied by using nanoindentation (NI). There was no microscopic crack or crack propagation in the YBCO scaffold structure of EPIP (Fig. 3A). The Olive–Pharr model was used to analyse the data obtained from the NI tests, it can be found that the YBCO scaffold and EP component of EPIP is stable against penetration, concurrently undergoes losses in its elastic modulus and hardness as penetration depths increase (Fig. 3B and Supplementary Fig. S14), which was ascribed mainly to the gradual increase in the grain boundary density and EP molecules between the YBCO nanograins (Fig. 3C). YBCO single domain samples prepared by using the TSMTG process have high elastic modulus and microhardness due to the formation of large grain sizes during the long growth time (Supplementary Fig. S15). To assess the mechanical reliability of the EPIP sample, Vickers indentation tests were performed in ambient air on the polished surfaces. Although the radial cracks usually formed at the corners of the TSMTG imprints, there were no radial cracks nucleated at the corners of EPIP (Fig. 3D). Whether a larger or smaller pressure was applied to the YBCO scaffold of EPIP, the indentation of the YBCO skeleton part was visible and there was no crack or breakage (Supplementary Fig. S16). The hardness and indentation characteristics of each material are listed in Supplementary Table S3.

We now turn to discuss the macroscopic mechanical properties of EPIP, EP and TSMTG samples at 77 K by using a uniaxial compressive test. The stress–strain curves are displayed in Fig. 3E. One can see that the mechanical properties of EPIP are also superior to those of the TSMTG sample. In detail, the compressive strength of the TSMTG sample at 77 K is 212 MPa and the ultimate strain is 1.53%. The compressive strength of EPIP at 77 K is 412 MPa, which is ∼77 times that of the YBCO-3D sample and ∼1.94 times that of the TSMTG sample (Supplementary Fig. S17), respectively. In terms of toughness, the static toughness value of the YBCO-3D sample and the TSMTG sample at 77 K are 803 and 1426 kN m^−2^, respectively, while the static toughness value of EPIP is 10 112 kN m^−2^, which is 12.6 times that of the YBCO-3D sample and 7.1 times that of the TSMTG sample (Fig. 3F). The specific strength and specific stiffness of EPIP were greatly improved compared with those of the YBCO-3D and TSMTG samples (Fig. 3G). We also conducted three-point-bending tests of the three kinds of samples at 77 K. It can be seen from Supplementary Fig. S18 that the flexural modulus and strength of the EPIP material at 77 K are 13.4 GPa and 132.05 MPa, respectively. The bending strength and the ultimate failure strain of the EPIP material are 1.41 times and ∼10 times that of the TSMTG material at 77 K. As shown in Supplementary Fig. S19, the normalized fracture energy that reflects the toughness of the EPIP material is 638.6 kN m^−2^, which is 14.8 times that of the TSMTG sample (43.3 kN m^−2^). The macro mechanical data for the used samples obtained by using the flexural tests at room temperature and 77 K are listed in Supplementary Table S4. We find that no matter whether at room temperature or 77 K, EPIP has greater deformation ability than the TSMTG material. To assess the fracture morphology, we also conducted a uniaxial compressive process at room temperature. The total compressive process of the TSMTG, EPIP and YBCO-3D samples are recorded in Movie 2, Movie 3 and Movie 4, respectively. Moreover, the compressive stress–strain curves are shown in Supplementary Fig. S20 and the corresponding results are listed in Supplementary Table S5. As can be seen in Fig. 3H, a combined crack morphology with deflection, branching and crack blunting was observed in EPIP, while the long-range weak cleavage plane crack occurred in the TSMTG samples (Supplementary Fig. S20 and Movie 2). Microstructure characteristics and robustness analysis of the EPIP and TSMTG sample are displayed in Supplementary Fig. S21; one can conclude that EPIP exhibits better comprehensive mechanical properties and mechanical transfer efficiency.

### Superconducting properties of EPIP

**Figure 4.**
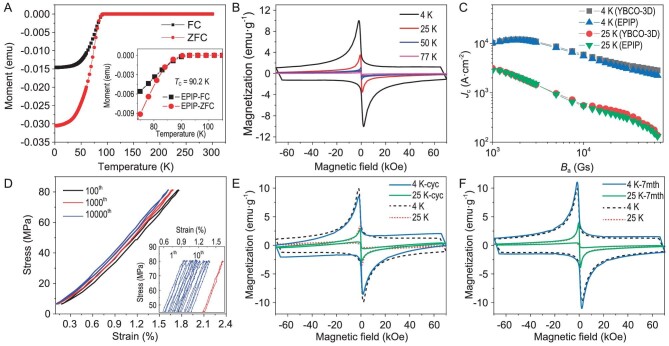
Electromagnetic properties of EPIP. (A) The zero-field-cooled (ZFC) and field-cooled (FC) curves of EPIP sample at an applied field of 100 Oe (inset shows the point of superconducting transition). (B) Magnetic hysteresis loops of EPIP sample at 4, 25, 50 and 77 K. (C) *J*_c_ (H) of 3D-printed YBCO with an increase in temperature from 4 to 25 K. (D) Stress–strain curves for fatigue testing (inset for TSMTG simple). (E) Magnetic hysteresis loops of EPIP after fatigue testing. (F) Magnetic hysteresis loops of EPIP exposed to air for 7 months.

The YBCO-3D, EPIP and TSMTG samples were cut into 4 × 2 × 2 mm^3^ for an electrical performance test. As shown in the inset of Fig. 4A and Supplementary Fig. S22, the critical temperature *T*_c_ of the YBCO-3D, EPIP and TSMTG samples are 89.9, 90.2 and 90.1 K, respectively. Simultaneously, compared with the YBCO sample prepared by TSMTG, the superconductivity of the EPIP sample has much space for improvement in critical current. Figure 4B shows the plot of magnetization versus magnetic field (from –70 to 70 kOe) of the EPIP sample at temperatures ranging from 4 to 77 K. As can be seen, a larger magnetic moment was obtained at a lower ambient temperature under the same intensity of the external magnetic field. Figure 4C shows the *J*_c_ (H) of EPIP and YBCO-3D, which decreases exponentially with the magnetic field in two samples at different temperatures (4 and 25 K). From these results, we can conclude that the presented toughening technology has no actual effects on the superconducting properties of the samples.

Figure 4D shows the EPIP sample stress–strain curves of with 100 cycles, 1000 cycles and 10 000 cycles, respectively. The EPIP material shows less plastic deformation and modulus degradation as the number of cycles increases (Supplementary Fig. S23), which implies stable mechanical properties. However, the TSMTG sample fractures after 25 cycles (illustration in Fig. 4D). After 10 000 cyclic loadings, the critical temperature of EPIP does not change significantly and remains constant at 90 K (Supplementary Fig. S24). The maximum magnetization of EPIP at 4 and 25 K are –8.57 and –2.83 emu g^−1^, respectively. Before cyclic loading, the maximum magnetization of EPIP at 4 and 25 K are 10.03 and –3.5 emu g^−1^, respectively. One can see that at 4 K the maximum magnetization is reduced by 14.6% (Fig. 4E). Generally, the YBCO materials suffer from functional impairment in long-term exposure to air due to hydrolysis reactions and carbonate contamination. As shown in Supplementary Fig. S25, after EPIP is exposed in a humid environment for 7 months, the *T_c_* is still ∼90 K. The magnetization of EPIP maintains 10.84 emu g^−1^ at 4 K (Fig. 4F). Therefore, the combination of EP and YBCO-3D forms a dense structure, since the epoxy resin prohibits the reaction of the YBCO component with water and carbon dioxide. This process eventually enhances the durability of the material.

## CONCLUSION

We demonstrated the creation of lightweight YBCO bulks that reached amazing toughness and durability. This was achieved using an interlocking dual network construction that is capable of deforming elastically and plastically via network interaction. These results serve to promote the applications of YBCO bulk superconductors. Tough and durable superconductors without superconductivity degradation represent the future pursuit of superconducting materials, where it is possible to transform a strong and brittle functional ceramic into a lightweight, tough and durable composite material without function degradation.

## MATERIALS AND METHODS

### Materials

Yttrium oxide (Y_2_O_3_ AR. 99.5%), barium carbonate (BaCO_3_ AR. 99.95%), copper oxide (CuO 99.5%) and carboxymethyl cellulose sodium (RnOCH_2_COONa, USP) with a viscosity of 600–1000 mPa were purchased from Shanghai Macklin Biochemical Technology Co., Ltd (China). Epoxidized soybean oil (C_57_H_98_O_12_ AR.) was obtained from Shanghai Aladdin Biochemical Technology Co., Ltd (China). Low-temperature epoxy resin was obtained from Composite Technology Development, Inc. (Lafayette, USA). Deionized water, ethanol, oxygen and liquid nitrogen were obtained from local suppliers (Lanzhou, China). All chemicals were used as received without any post-treatment or processing.

### Characterization

XRD analysis was performed using an X-ray diffractometer (X’PERT PRO MPD, PANalytical, Netherlands) with Cu Kα radiation in the 2θ range of 8°–80°. SEM (HELIOS NanoLab 600i, FEI, US) and transmission electron microscope (TEM) (Tecnai G2 F30, FEI, US) were performed to analyse the morphology and microstructures of the samples. EBSD was conducted using an OPTIMUS TKD detector (Bruker QUANTAX EBSD, GER) Thermogravimetric analysis (TGA) was performed from 25°C to 1000°C at a heating rate of 10°C/min using a simultaneous TGA/DSC thermal analyser (TGA/SDTA851e, Mettler-Toledo, Switzerland). Atomic Force Microscope (AFM) used the plug-in that comes with nanoindentation.

### Measurements

Nanoindentation was tested by using the triangular pyramid diamond of the Berkovich indenter (American Hysitron TI-950 Nanoindenter); microhardness was measured by using a Vickers Hardness Tester (Matsuzawa, MMT-X BRUCK Vickers, JPN). The sample dimensions were measured using a Vernier caliper (BK-318, BIAOKANG, China). The mechanical properties were studied using an Instron 4505. Fatigue tests were conducted using an INSTRON 8802 fatigue machine. Magnetization measurements were performed using a SQUID magnetometer (MPMSR2, Quantum Design). The magnetic moment was measured as a function of the temperature with an applied magnetic field of 100 Oe under zero-field-cooled (ZFC) and field-cooled (FC) conditions. Magnetic hysteresis loops were measured at temperatures of 4, 25, 50 and 77 K under applied fields ranging from –70 to 70 kOe.

## Supplementary Material

nwad030_Supplemental_Files
