# Supplementary material for: Lightweight, highly tough and durable YBa2Cu3O7–x superconductor
Source: Natl Sci Rev. 2023 Feb 10;10(3):nwad030. doi: 10.1093/nsr/nwad030 (PMC10062695; doi:10.1093/nsr/nwad030)
Supplement: nwad030_Supplemental_Files [file nwad030_supplemental_files.zip › Supplementary_Materials.docx]

Supplementary Materials for

**Lightweight, highly tough and durable YBa2Cu3O7–x superconductor**

**Baoqiang Zhang1,2, Xingyi Zhang1,2,* and You-He Zhou1,2**

1Key Laboratory of Mechanics on Disaster and Environment in Western China attached to the Ministry of Education of China, Lanzhou University, Lanzhou, Gansu 730000, PR China

2Department of Mechanics and Engineering Sciences, College of Civil Engineering and Mechanics, Lanzhou University, Lanzhou, Gansu 730000, PR China

*Corresponding author. Email: zhangxingyi@lzu.edu.cn

**TABLE OF CONTENTS**

**This word file includes:**

S1: 3D-Printing YBCO

S2: Preparation of composite YBCO superconductors (EPIP)

S3: Mechanical properties

S4: Superconducting properties

Figs. S1 to S25

Tables S1 to S5

**Other Supplementary Materials for this manuscript include the following:**

Movies S1 to S4

## S1. 3D-Printing YBCO


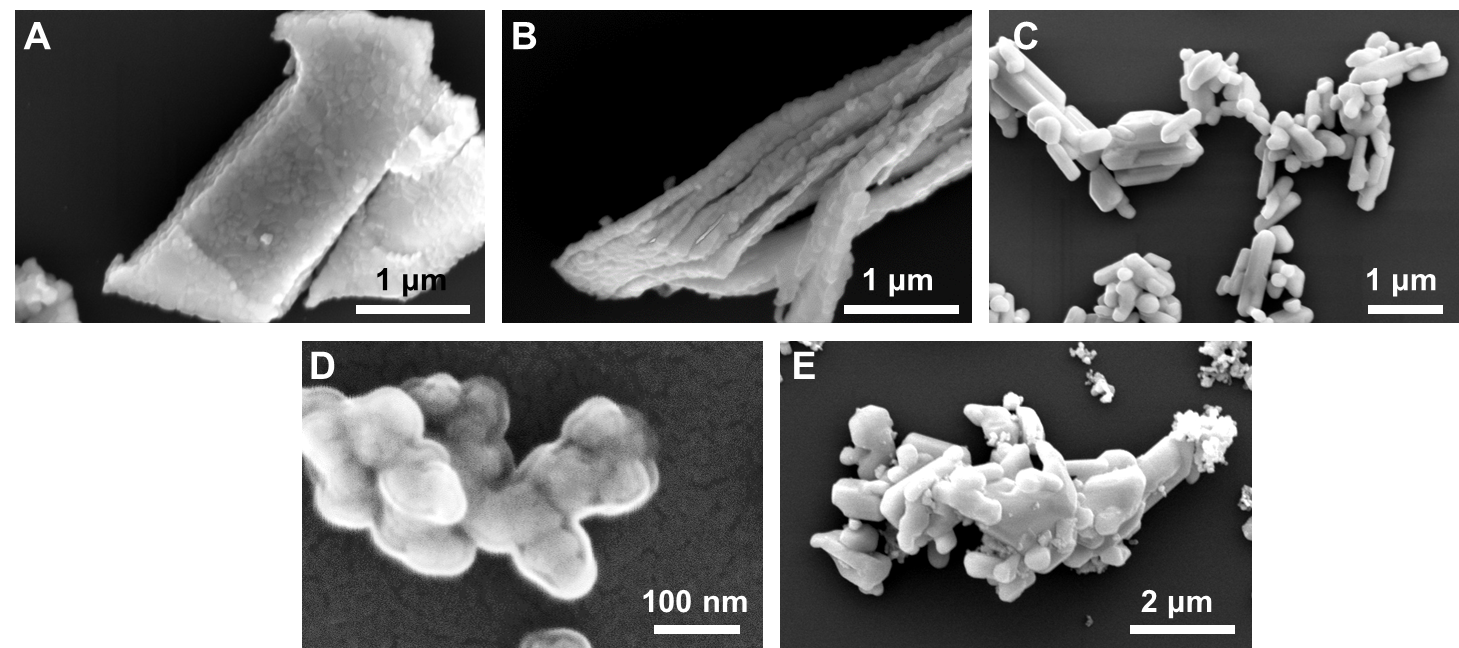


**Fig. S1.** SEM images of YBCO precursor grains. (**A**) Y2O3 surface. (**B**) Y2O3 lamellar structure of cross sections. (**C**) BaCO3 stick-like shape. (**D**) CuO nanoparticles. (**E**) Aggregate accumulation form of the mixtures.

As shown in **Fig. S1 A** and **B,** Y2O3 is the loose and stacked lamellar structure. BaCO3 is short rod structure and CuO is spherical nanoparticles (**Fig. S1 C**-**E**).

The mixture precursor powder by a stoichiometric molar ratio of 1:2:3 for Y2O3 (40g), BaCO3 (139.8g) and CuO (84.4g) was prepared. 160 ml ethanol was mixed with YBCO precursor powders and conducted 10 min ball milling treatment to make the Y2O3 layers were exfoliated and well-mixed with other particles. After the ball milling, the slurry removed the ethanol solvent becoming dispersed powder at 80 ℃ for 24 h.

Then, the aqueous solution of carboxymethyl cellulose sodium with a mass fraction of 6.25% was prepared. In addition, to further improve the printability of YBCO precursor，the soybean oil epoxide was well mixed with the aqueous solution of carboxymethyl cellulose sodium in a mass ratio of 1:4. The water-oil mixed solution was added into dispersed powder by weight ratio of 2:3. After mechanically stirring for 30 min, the aqueous solution becomes viscous and gradually gels, showing elastic characteristics. In brief, the YBCO precursor powder mixture was mixed with carboxymethyl cellulose sodium, epoxidized soybean oil, and water in a weight ratio (w/w) of 1:0.033:0.133:0.5 and homogenized to prepare the printable paste.In order to obtain the homogeneous paste, the mixture was homogenized by roller milling.


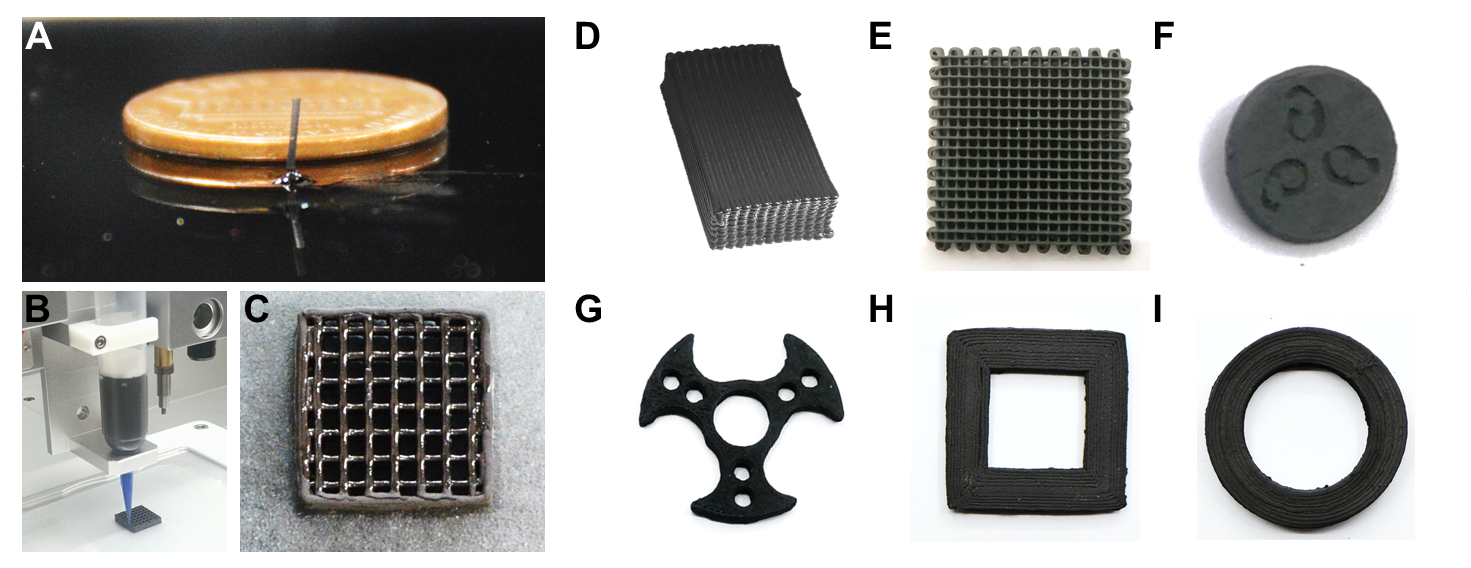


**Fig. S2.** 3D printing process and completed samples.(**A**) The plastic properties 3D printing filament with diameter of 200 μm. (**B**) Optical image of apparatus used. (**C**)Photographs of as-prepared YBCO precursor green body. (**D-I**) The printed YBCO samples with different structural features.

As shown in **Fig. S2A**, free standing vertical line by printable YBCO precursor paste. As shown in **Fig. S2B, C,** 3D frame lattice structure of the wet YBCO precursor bulk in the vertical direction that shows the high viscosity and shape retaining ability of paste for 3D printing. In **Fig. S2D-I**, the 3D structures were elaborately constructed by a continuous layer-by-layer deposition process. All the samples retain the high aspect ratio configuration and no shrinkage in the plan.


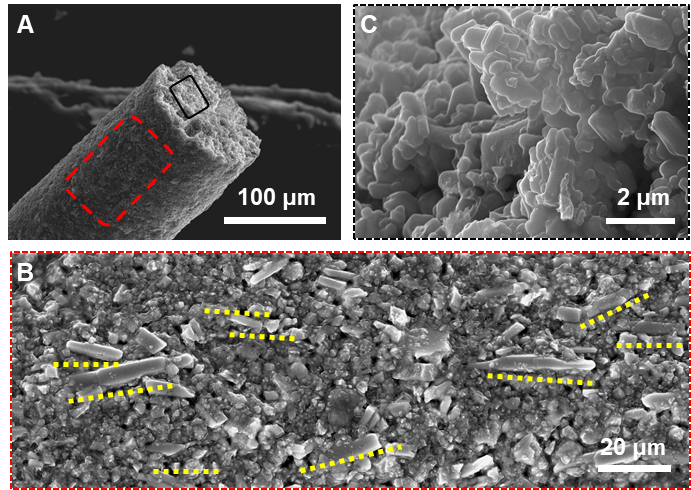


**Fig. S3.** (**A** and **B**) SEM images of the extrused round rod paste show the alignment of the Y2O3 and BaCO3 along the fiber direction before the sintering. (**C**) SEM image of the cross section of round rod.

The designed structures were created by a 3D-Bioplotter (3D-Bioplotter® Manufacturer series. Envision TEC.) through a layer-by-layer deposition procedure of paste. To obtain quasi-isotropic uniform and ordered robust a lamellar skeleton structure and to prevent possible shrinkage during fabrication, the printed wet samples were three-dimensional freezing cold casting manipulating microstructure. The freezing procedure that the printed wet YBCO bulk was placed in - 45 ℃ n-hexane solution for 3min and then put on the suspended support in - 45 ℃ environment for 24h was carried out. It promoted intimate contact and ordered orientation among the precursor particles under the directional squeezing of the ice crystals. This reduced the perforation cracks, severe shrinkage, and spatial anisotropy of the 3D-printed YBCO green body during the subsequent drying and sintering processes. Finally, the freeze-drying green body was sintered combining with oxygenation to obtain superconducting YBCO samples. The total process of 3D printing is schematized in **Fig. S4**.


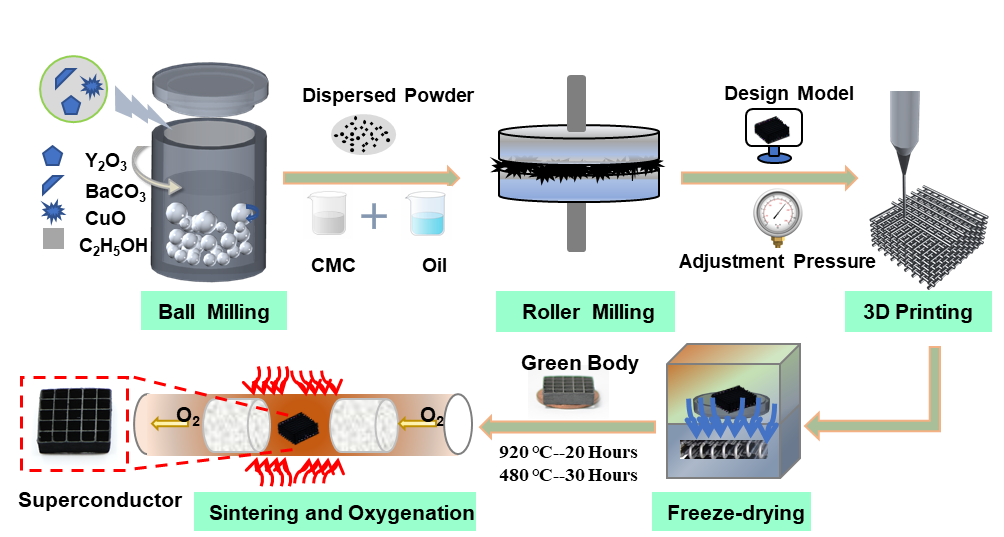


**Fig.S4.** Schematic of fabrication of YBCO superconductor by 3D-printing followed by freeze-drying and annealing.

## S2. Preparation of composite YBCO superconductors (EPIP)

Epoxy resin (CTD-101K, denoted as EP) is selected as the reinforcing phase, and the vacuum pouring process is used to compound the reinforcing phase to realize the reinforcement and toughening of YBCO superconducting bulk. The specific process flow is shown in **Fig. S5**. Firstly, the YBCO superconducting bulk with design structure is prepared by ceramic 3D printing method. The three-dimensional staggered structure of 3D printed YBCO was composed by tablet ribs (**Fig. S5**). Prepare the liquid epoxy resin reinforced toughened phase according to the proportion, and mechanically stir for 5min until it is evenly mixed. Then, the liquid epoxy resin toughened phase was heated to 105℃, the prepared YBCO superconducting bulk was placed in the liquid, and the reinforced and toughened phase was injected into the YBCO superconducting bulk in the way of self-continuous flow in the vacuum environment. There are two mainly factors affect the density of EPIP in the production of impregnation. One is the heating temperature. As seen in Fig.S6, when the temperature was high than 100 °C, the density of the EPIP obtained stabilized compactness under a vacuum degree. Therefore, the immersion temperature was used at 105°C, which slightly higher than the stable temperature. The other one is vacuum degree. According to the experiment, low vacuum degree will cause the boiling of liquid epoxy resin, and high vacuum degree will not have enough pressure to make the liquid epoxy resin fill the skeleton of 3D printing bulk. Therefore, we adopt an optimal vacuum degree that is about 100 mbar, which can obtain a stable state. Finally, the composite system was heated and cured at 125℃ for 12h to form a dense YBCO superconducting complex with high strength and toughness.


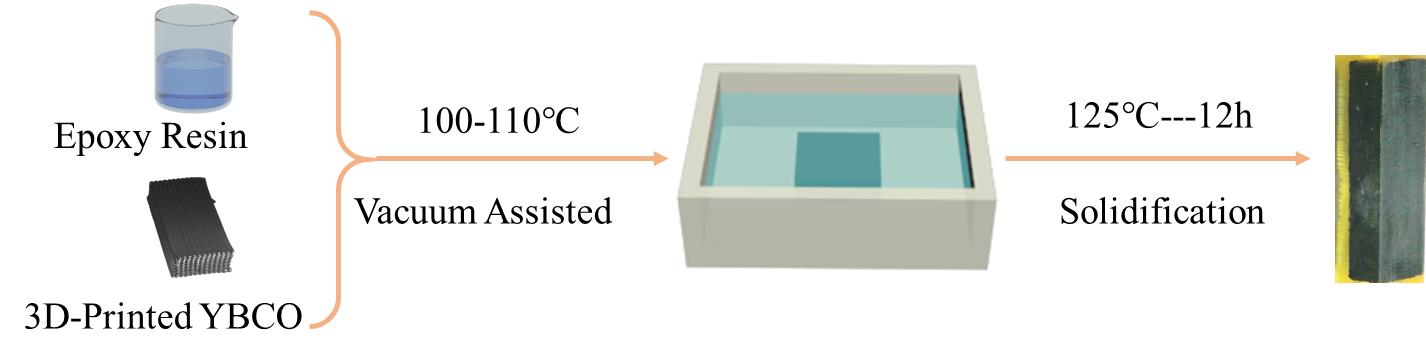


**Fig. S5.** Preparation scheme of strengthened and toughened superconducting bulk materials


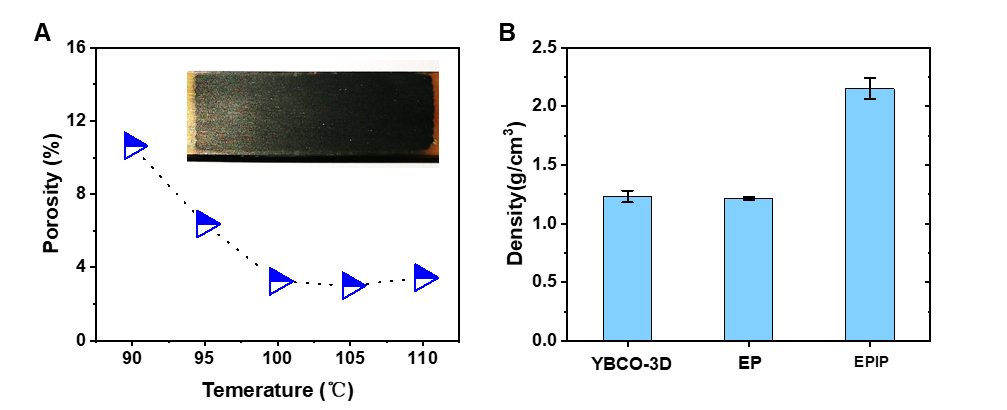


**Fig. S6** (**A**)The relationship between impregnation temperature and compactness. (**B**)The density of different materials.

The densities of composite samples containing various material compositions. Among them, the density of YBCO-3D is about 1.25 g/cm3, the density of cured EP is 1.21 g/cm3, the density of EPIP is 2.15 g/cm3, and the porosity is calculated as follows:

For 3D printed YBCO bulk:

（1）

（2）

（3）

Among them, the apparent volume of the 3D printed sample; the mass of the 3D printed YBCO sample; the apparent density of the 3D printed material; the absolute volume of the YBCO superconducting material; the porosity of the 3D printed sample; the absolute density of the YBCO superconducting material 6.38 g/ cm3.

For epoxy cured materials, the epoxy can be considered fully dense when cured:

（4）

Among them, the volume of epoxy resin; the mass of epoxy resin. Density of epoxy resin.

For composite materials, which contain YBCO components and epoxy resin components, the equivalent density calculation formula is as follows:

（5）

（6）

Among them, the apparent volume of the composite material; the mass of the composite material; the apparent density of the composite material; the porosity of the composite material. Finally, the minimum porosity of the EPIP is 3.2%. The density of YBCO bulks prepared by different methods is shown in **Table S1**


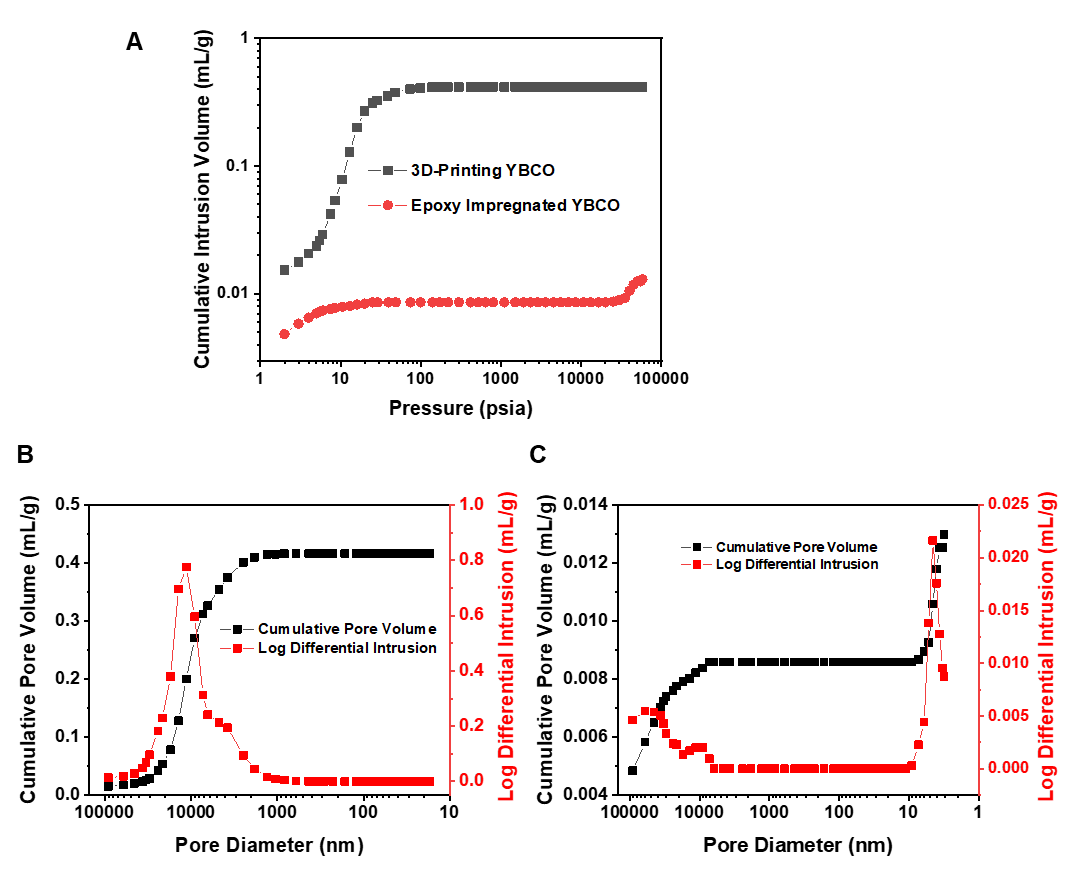


**Fig. S7.** (**A**)Cumulative mercury intrusion vs. applied pressure curve of YBCO-3D and EPIP materials measured by mercury intrusion porosimetry (MIP); Cumulative pore volume and differential intrusion distribution curves measured by the mercury intrusion test. (**B**) YBCO-3D. (**C**) EPIP.

The apparent skeleton density of YBCO-3D was 5.39 g cm-3, the porosity was 69.2%, and the specific surface area was 0.213 m2 g-1.


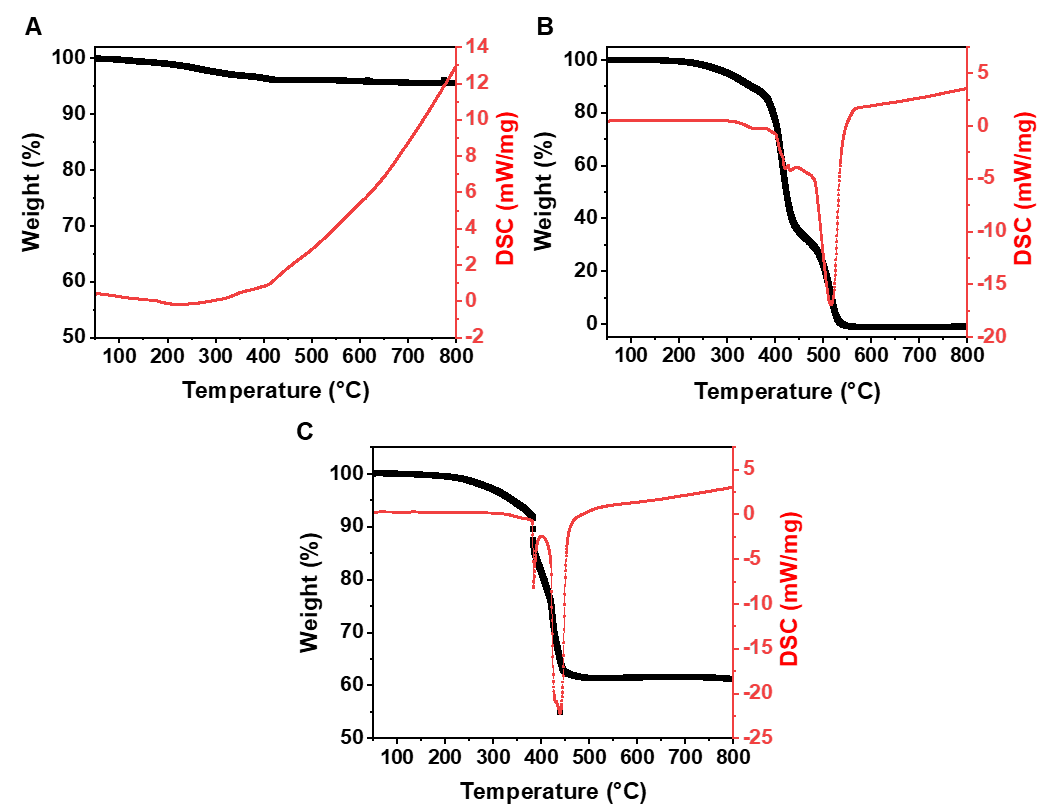


**Fig. S8.** TGA and DSC date of different materials(**A**) YBCO-3D. (**B**) EP (CTD-101K). (**C**) EPIP

The weight loss of the EPIP decreases sharply after heating up to 382 ℃. When the temperature rises to 471 ℃, the weight loss was almost no longer decreased and the loss mass value was 39% simultaneously. Thus, the ratio of the epoxy resin mass immersed in the YBCO bulk skeleton to YBCO component was 39:61. The porosity of the composite calculated from the above compactness is 3-4%. Therefore, it can be calculated that the porosity of YBCO skeleton is about 77%.


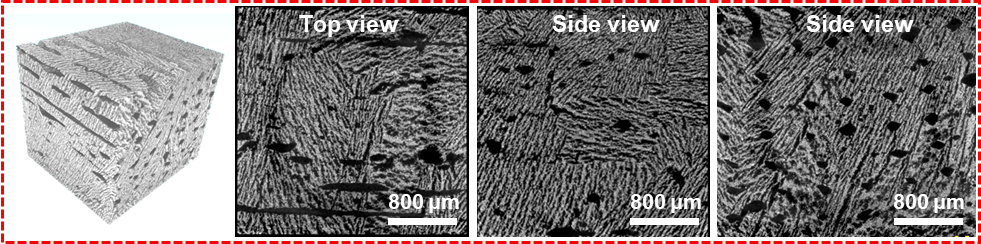


**Fig. S9.** 3D reconstruction of toughened YBCO derived from X-ray microtomography, and microtomography images display the uniform, different orientation pores.


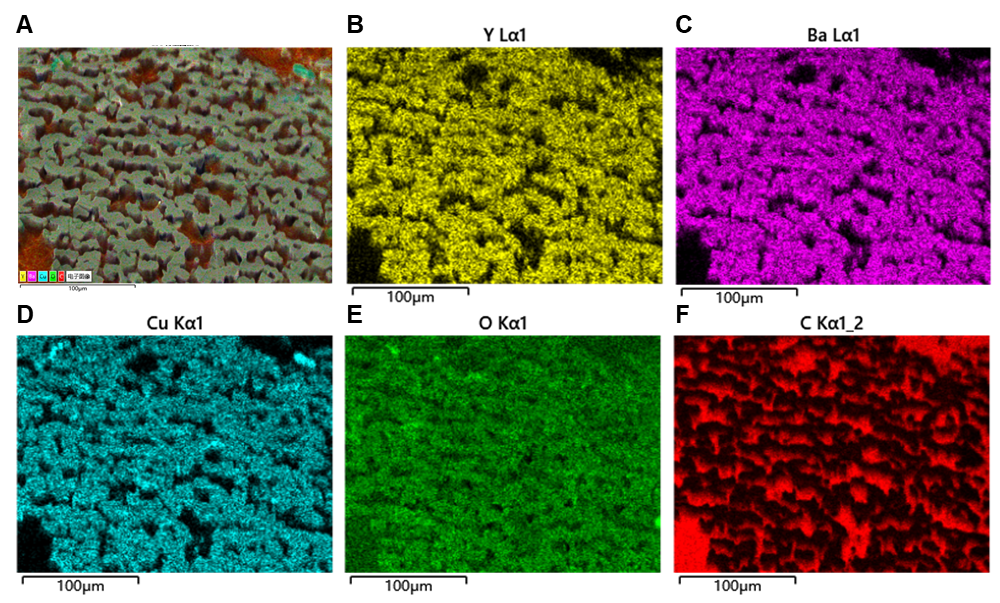


**Fig. S10.** EDS elemental mapping images of EPIP sample.(**A**) Full overlay element. (**B-F**) elemental mapping (Y, Ba, Cu, O and C).

**
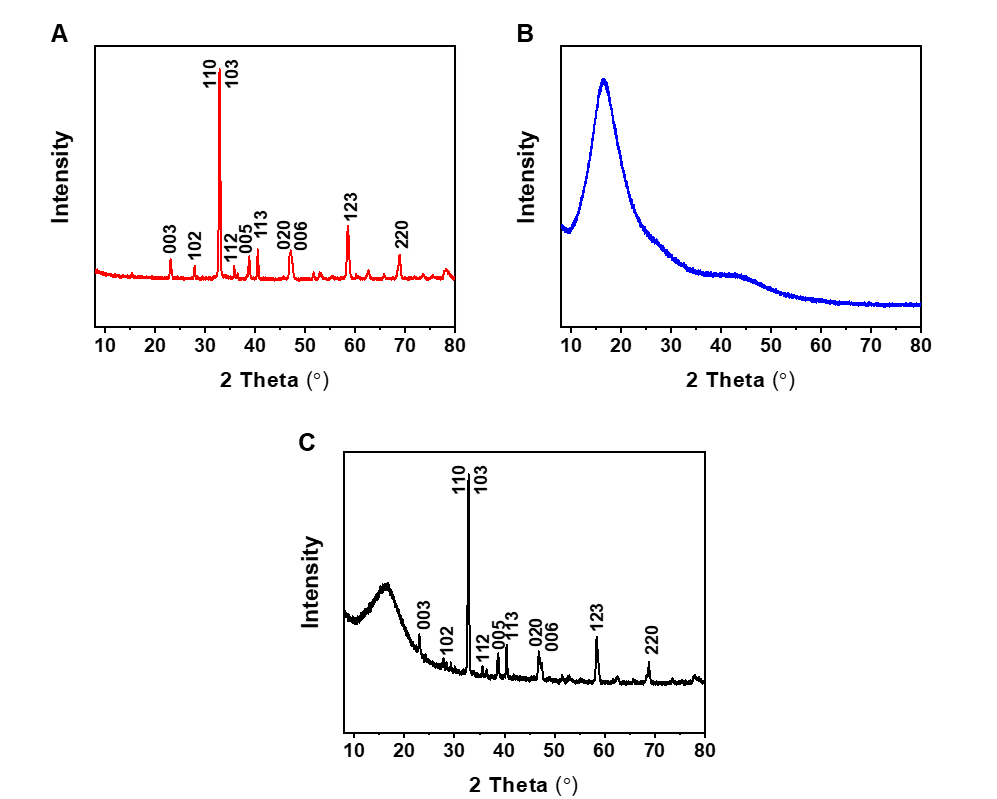
**

**Fig**. **S11**. XRD patterns. (**A**) EPIP. (**B**) YBCO-3D. (**C**) EP (CTD-101K).

**
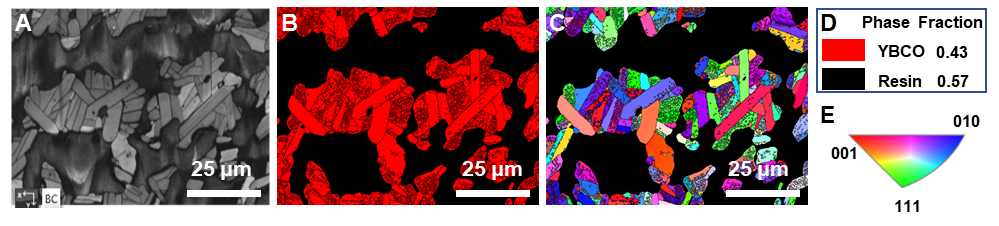
**

**Fig.S12**. **(A)** The band constrast map of EPIP. (**B**) Phase map and relative phase fraction. (**C**) 001 IPF orientation map for the Y123 phase. (**D**) color bar for image (**B**). (**E**) color scale for image (**C**).

**
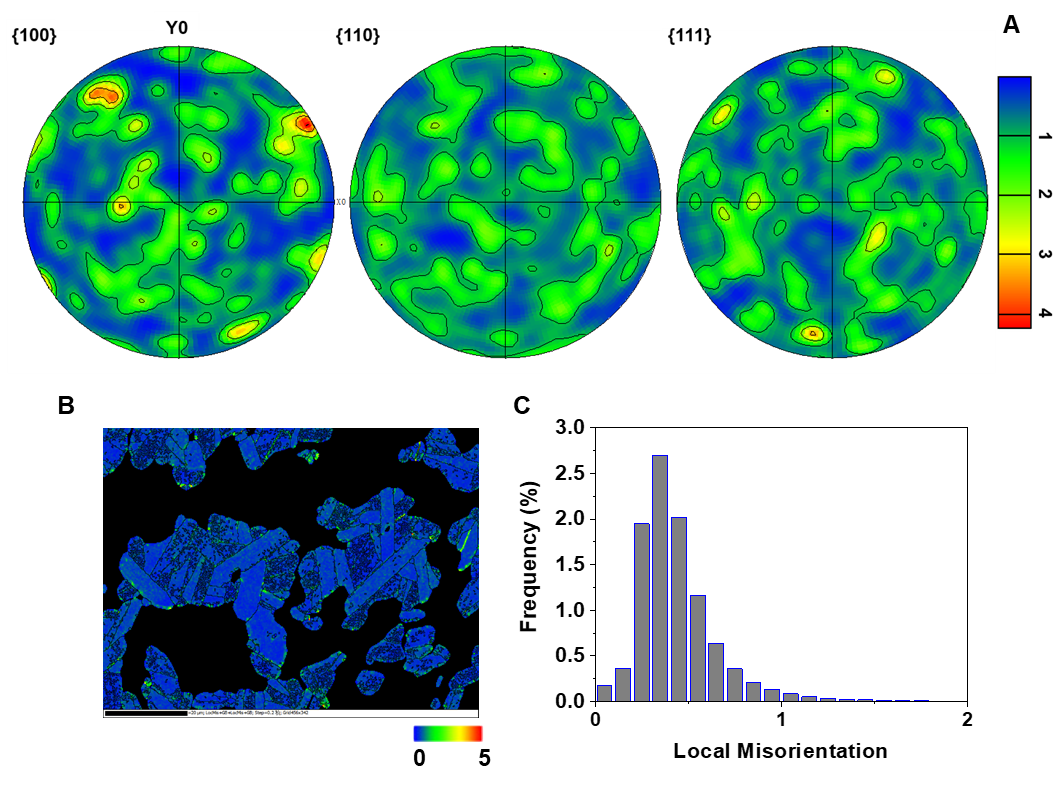
**

**Fig. S13. (A**)The corresponding pole figure distribution, **(B)** The corresponding KAM map and **(C)** Local Misorientation distribution.

## S3. Mechanical properties


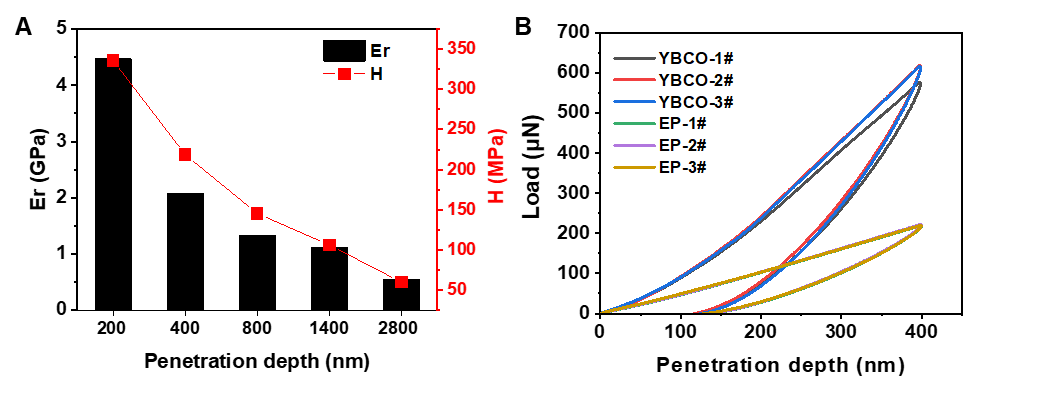


**Fig. S14**. Nanoindentation test of EPIP. (**A**) The relationship of different penetration depth between with modulus and hardness. (**B**) Penetration depth-load curves of YBCO skeleton and epoxy resin component in EPIP.


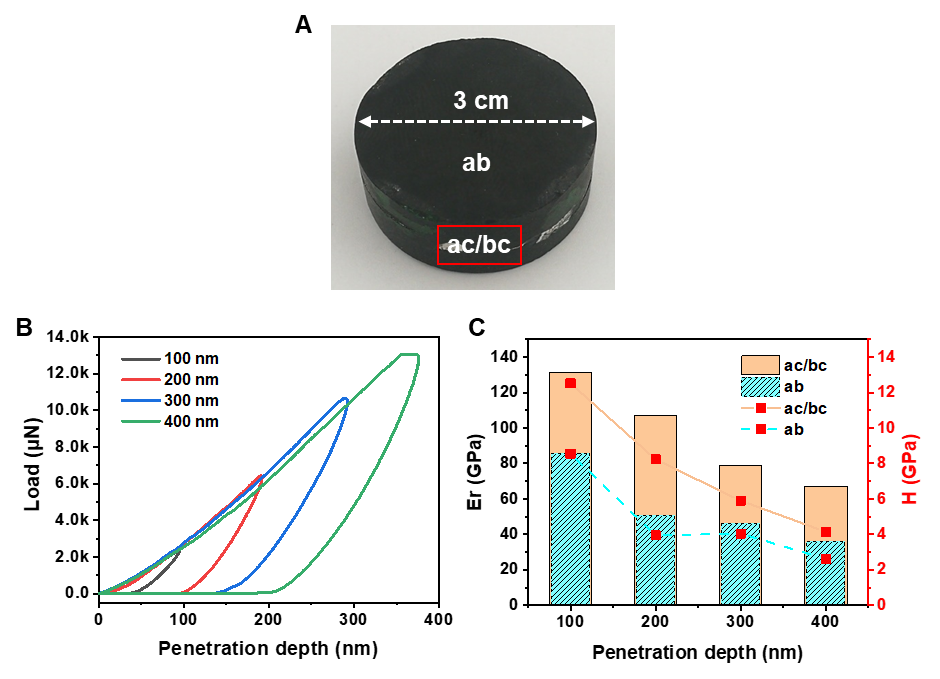


**Fig. S15.** Nanoindentation test of TSMTG.(**A**) Optical photo of single domain YBCO bulk prepared by TSMTG. (**B**) The Load-displacement curve of TSMTG. (**C**) Microscopic elastic modulus and hardness of crystalline section of TSMTG.

**Top-seeded melt textured growth (TSMTG) process for YBCO single domain superconductors**

Its specific preparation process and conditions are as follows:

1. YBCO precursor powders were prepared from Y2O3, BaCO3 and CuO powders with the ratio of Y: Ba: Cu = 1.8:2.4:3.4 in sintering furnace by a solid state reaction. For Y211s preparation, the mixed powder was calcined in air twice at 950℃ for 24 h. In order to refine the 211 phase 0.2 wt.% of Pt powder was added.

2. The precursor powders were mixed thoroughly with a nominal composition of Y123:Y211 = 1: 0.4 in a molar ratio. Well mixed powders were pressed into pellets 35 mm in diameter and 22 mm in thickness with uniaxial mould pressing.

3. SmBaCuO seeds were used to predetermine the orientation of the growing YBCO single grains. One single seed was applied on the top of the cylindrical shaped disks. The c-axis of the seeds was perpendicular to the top face of the disks.

4. Y-Ba-Cu-O bulk samples are prepared by the melt-textured-growth method, associated with the top seeded technique. In brief, the precursor with SmBaCuO seeds are placed into a crucible furnace and heated to 1040℃ for 3 h, held for 60 min, cooled down to 1000℃ rapidly, and then slowly cooled down to 980℃ with a rate of 0.5℃/h, and finally furnace cooled to room temperature.

5. The bulks oxygenated at 500-450 ℃ in a tubular furnace with flowing oxygen for 100 h.


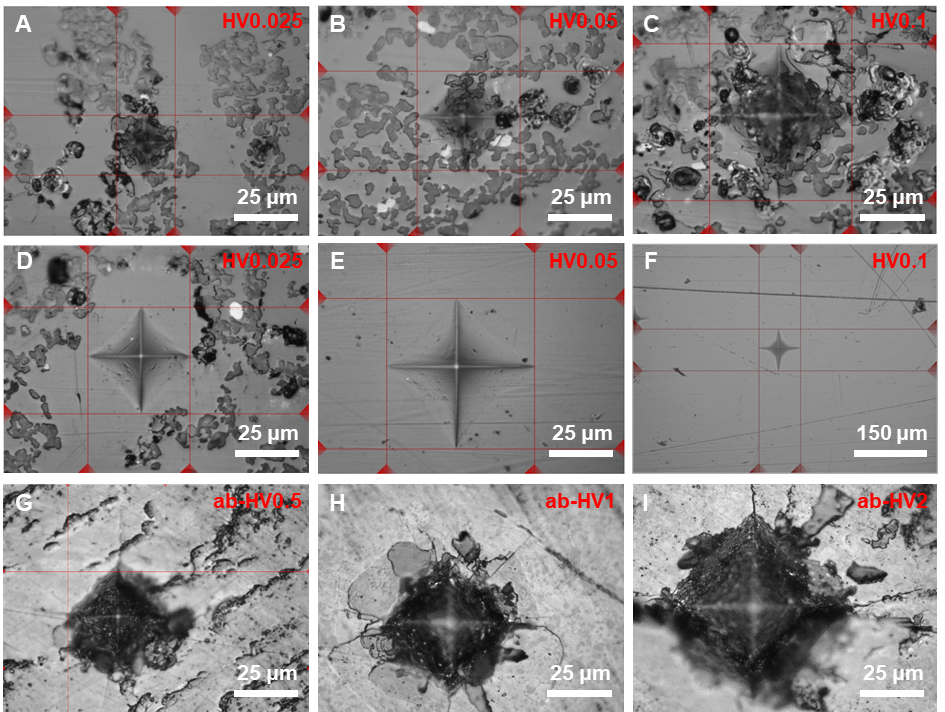


**Fig. S16.** Optical micrograph of Vickers indentation introduced on a polished section with different loads.(**A-C**) YBCO components of EPIP. (**D-F**) epoxy resin components of EPIP. (**G-I**) ab crystal plane of TSMTG.


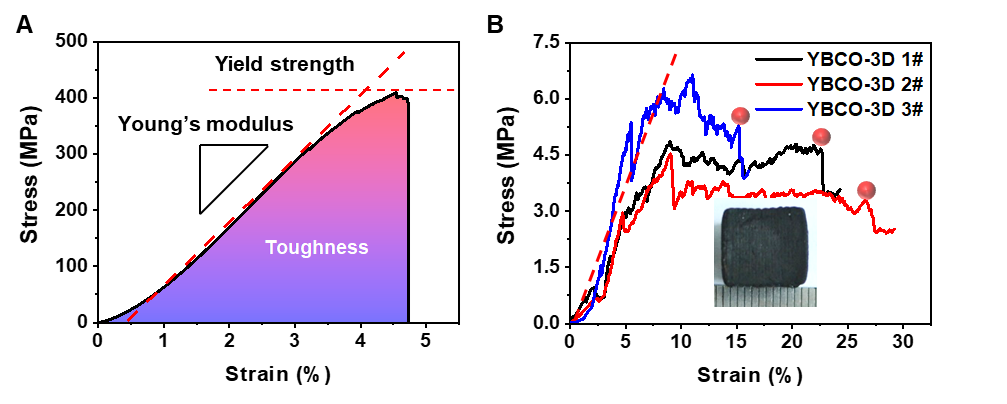


**Fig. S17**. Uniaxial compression test at temperature of 77K. (**A**) stress-strain curves of EPIP. (**B**) stress-strain curves of YBCO-3D. (YBCO-3D 1#, YBCO-3D 2#, YBCO-3D 3#, there are three different samples in the same case)

The area enclosed by the stress and strain curves under uniaxial compression is the static toughness of the material, which can reflect the toughness of the material. Fig. S17B shows the stress-strain curve of YBCO-3D bulks at temperature of 77K. The toughness calculation in the figure starts from the point of strain 0 and ends at the point where the stress drops suddenly, which has been marked with red dots in the figure. Three groups of experiments were conducted in this experiment, and the experimental samples were numbered YBCO-3D 1#, YBCO-3D 2# and YBCO-3D 3#, respectively.


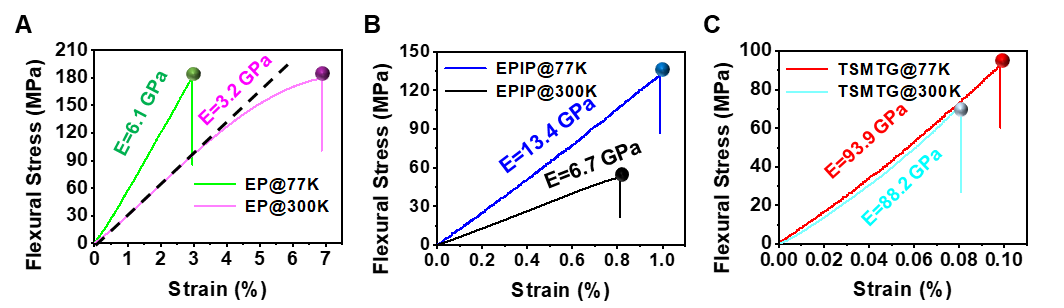


**Fig. S18**. Flexural stress-strain curves of materials with different temperatures. (**A**) EP. (**B**) EPIP. (**C**) TSMTG.


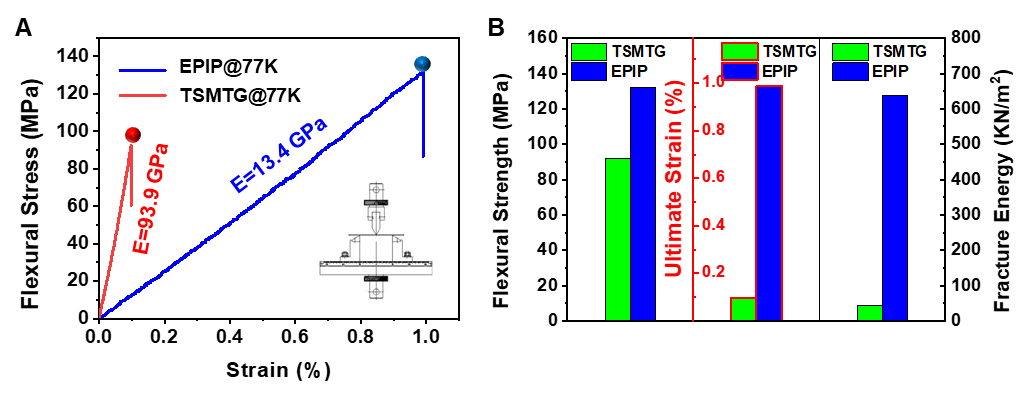


**Fig. S19.** Comparision of three-point bending test.(**A**) Flexural stress-strain curves of EPIP and TSMTG at temperature of 77K. (**B**) Comparison of flexural strength, ultimate strain and fracture energy of EPIP and TSMTG materials.

The EP, EPIP and TSSG materials were tested by three-point bending. The samples were cut to the size of mm3, and the test span was 24 mm. According to the plane assumption of bending deformation, the bending strength (MPa), bending strain (mm/mm), bending modulus of elasticity (MPa) and fracture energy (J/m2) are calculated as follows:

(7)

(8)

(9)

(10)

Where is the load (N); is the span between two supporting points (mm); is the width of the sample (mm); is the height of the sample (mm); is the mid span deflection/displacement (mm); is the maximum strain when the sample breaks (mm/mm). The normalized fracture energy is divided by the test span L, and the unit is KN/m2


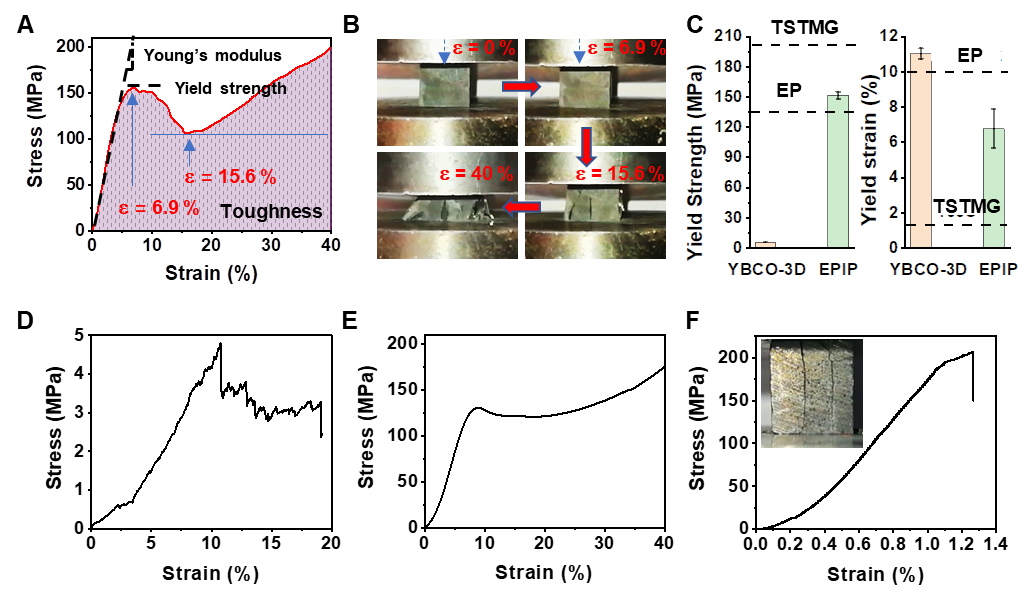


**Fig. S20.** Uniaxial compression test at about temperature of 300K**.** (**A**) EPIP. (**B**) The experimental snapshots of cross-sectional views under Uniaxial compression. (**C**) Comparison of yield strength and yield strain YBCO-3D and EPIP. (**D**) YBCO-3D. (**E**) EP(CTD-101k). (**F**) TSMTG


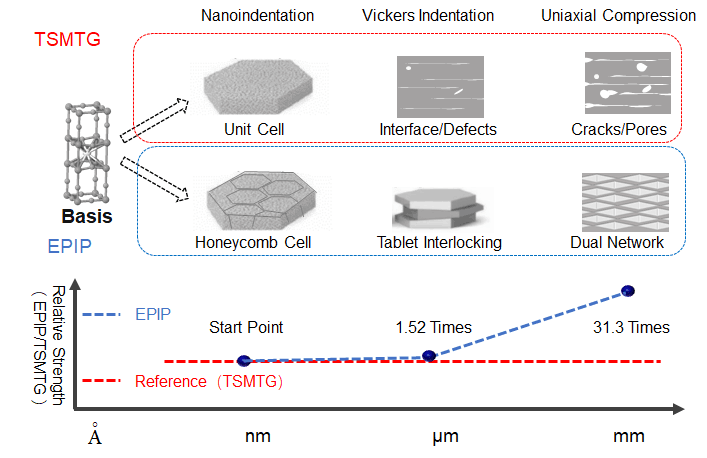


**Fig. S21**. **Microstructure characteristics and robustness analysis of the used samples.**

## S4. Superconducting properties.

**The calculation the critical current density (*J*c, A cm-2)**

The critical current density (*J*c, A cm-2),which is an important assessment index for the superconducting performance, was calculated from the *M-B*acurves using the Bean model formula:[33]

（1）

where ∆𝑀 is the width of the magnetization hysteresis loop (emu cm-3), and *a* and *b* (*a*>*b*) are the cross-sectional dimensions of the sample perpendicular to the field. As shown in Figure 5c, the *J*c (*H*)exponentially decreased with increasing magnetic ﬁeld at different temperatures (4 and 25 K). The double logarithmic plot of *J*c (*H*) demonstrated a nearly linear relationship with magnetic ﬁeld (1000–70000 Gs) without the so-called ﬁshtail effect.


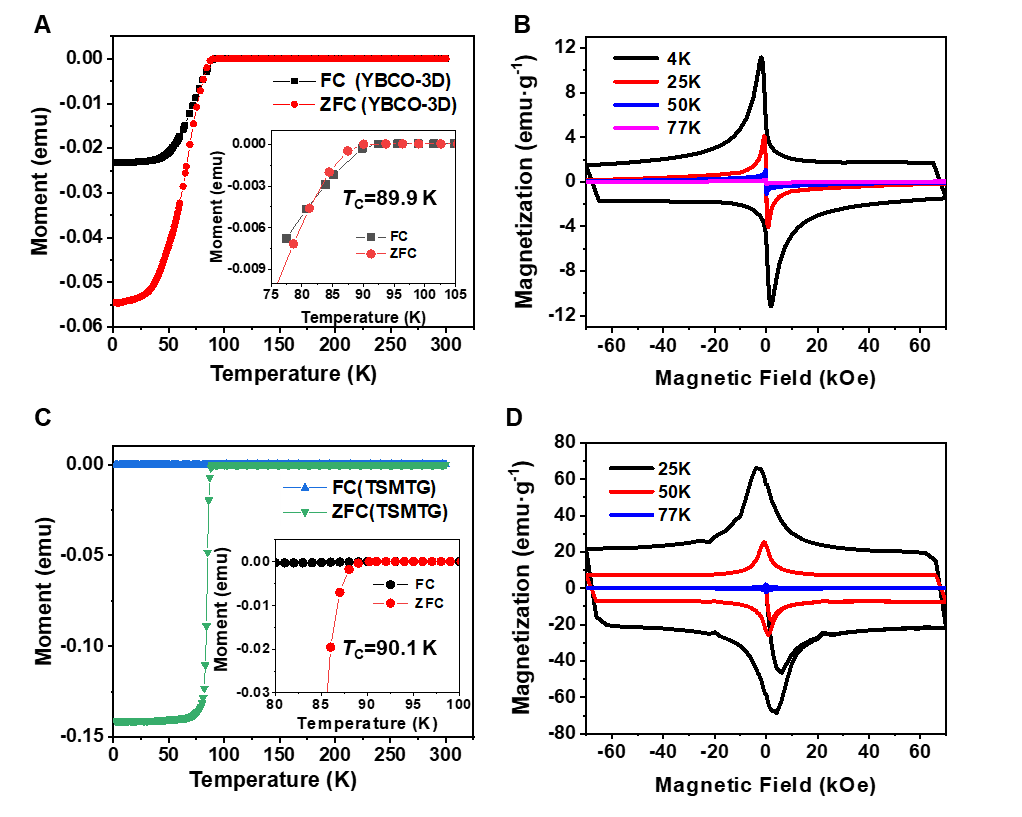


**Fig. S22.** Electromagnetic properties. (**A**) ZFC and FC curves of YBCO-3D sample at an applied field of 100 Oe (inset shows the point of superconducting transition). (**B**) Magnetic hysteresis loops of YBCO-3D sample at 4, 25, 50, and 77 K. (**C**) ZFC and FC curves of TSMTG sample. (**D**) Magnetic hysteresis loops of TSMTG sample.

**Fig. S23.** The variation value of elastic modulus with different cycles.


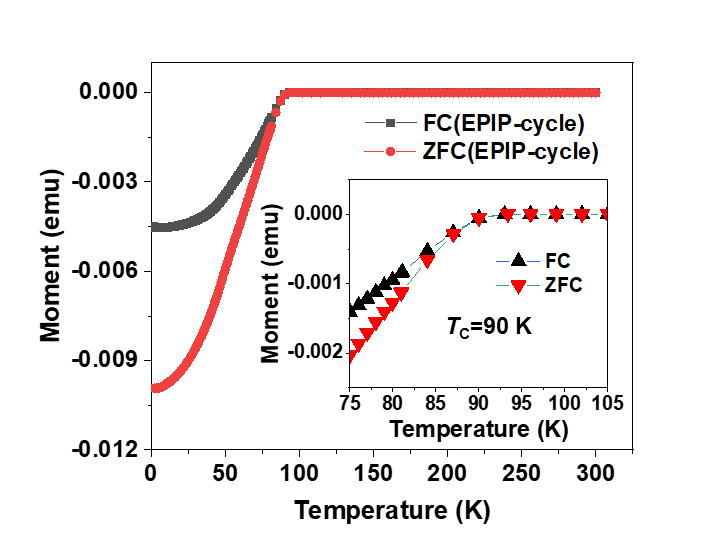


**Fig. S24**. ZFC and FC curves of EPIP sample after 10 thousand cycles of loading (inset shows the point of superconducting transition).


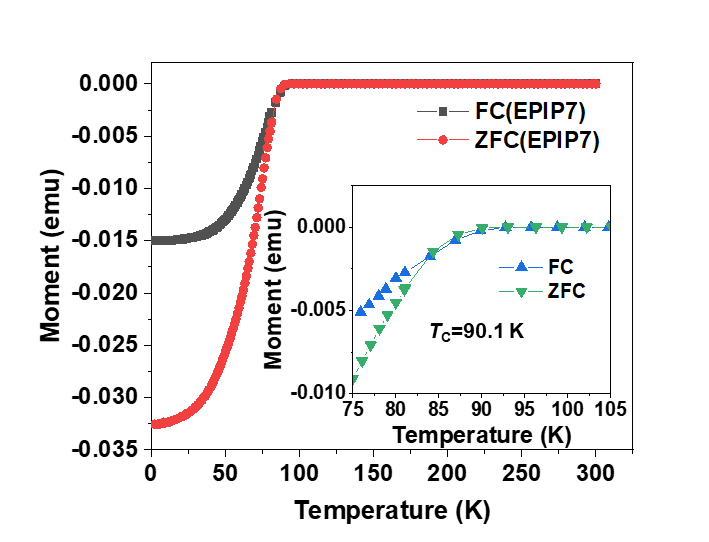


**Fig. S25.** **ZFC and FC curves of EPIP sample exposured to moisture after 7 months (inset shows the point of superconducting transition).**

**Table S1 Comparative density of YBCO samples fabricated via different methods.**

| **Fabrication method** | **Porosity(%)** | **Density**  **(g/cm3)** | **Critical temperature**  **(K)** |
| --- | --- | --- | --- |
| Solid state reaction[1] | 35 | 4.15 | 90 |
| Hot pressing [2] | 20 | 5.1 | 82.6 |
| Modified nitrate route[3] | 15 | 5.423 | ----- |
| C-YBCO composite superconductors[4] | 12~14 | 5.55 | 89±1 |
| Melt-textured growth (MTG)[5] | Almost 0 | 6.38 | 92 |
| Melt-textured growth(MTG) [6] | 10 | 5.74 | 91~93 |
| Top-seeded infiltration and growth (TSIG)[7] | 5 | 6.07 | 91 |
| Melt-textured bulks [8] | 6 | 6.01 | 91.7-92.1 |
| This work | 3.2 | 2.15 | 90.2 |

**Table S2. The pore size distribution and physical properties of the tested species from mercury intrusion porosimetry**

| **Sample** | **Total pore area**  **(m²/g)** | **Median pore diameter**  **(nm)** | **Average pore diameter**  **(nm)** | **Bulk density**  **(g/mL)** | **Apparent density**  **(g/mL)** | **Porosity**  **(%)** |
| --- | --- | --- | --- | --- | --- | --- |
| **YBCO-3D** | 0.213 | 11,068.20 | 7,822.64 | 1.6619 | 5.3978 | 69.211 |
| **EPIP** | 3.989 | 45,696.78 | 13.02 | 1.9531 | 2.0039 | 2.5358 |

**Table S3. Hardness testing of different sample by Vickers indenter**

| Sample | Position | Hardness scale  （Kg） | Hardness  （MPa） | Indentation diameter1  (μm) | Indentation diameter2  (μm) |
| --- | --- | --- | --- | --- | --- |
| TSMTG | ab section | HV0.5 | 718.99 | 33.8 | 38 |
| TSMTG | ab section | HV1 | 743.67 | 49.8 | 50.1 |
| TSMTG | ab section | HV2 | 703.08 | 71.5 | 73.7 |
| TSMTG | ac/bc section | HV0.5 | 900.87 | 32.2 | 32.0 |
| TSMTG | ac/bc section | HV1 | 906.59 | 45.45 | 45.05 |
| EPIP | YBCO section | HV0.025 | 80.58 | 23.7 | 24.2 |
| EPIP | YBCO section | HV0.05 | 56.2 | 41.3 | 39.9 |
| EPIP | YBCO section | HV0.1 | 50.35 | 61 | 60.4 |
| EPIP | Epoxy section | HV0.025 | 28.7 | 41.5 | 38.9 |
| EPIP | Epoxy section | HV0.05 | 25.81 | 59.4 | 60.4 |
| EPIP | Epoxy section | HV0.1 | 25.9 | 84.4 | 84.9 |

**Table S4. Macrocopic mechanical date for three-point-bending.**

|  | At 77 K temperature | | |  |  | At 300 K temperature | | |
| --- | --- | --- | --- | --- | --- | --- | --- | --- |
|  | **EP** | **EPIP** | **TSMTG** | | **EP** | | **EPIP** | **TSMTG** |
| **Flexural Strength**  **(MPa)** | 189.2±12.5 | 122.9±8.6 | 83.42±16.4 | | 176.8±2.25 | | 54.5±2.5 | 63.89 ±7.14 |
| **Fracture Energy**  **(KN m-2)** | 2870.2±395 | 532.4±94.5 | 35.66±11.9 | | 8172±992 | | 231.4±18.6 | 27.39±4.85 |
| **Ultimate Strain**  **(%)** | 3.06±0.15 | 0.9±0.08 | 0.088±0.018 | | 7.32±0.39 | | 0.84±0.05 | 0.084±0.05 |
| **Flexural Modulus**  **(GPa)** | 6.17±0.1 | 13.7±0.44 | 94.48±0.52 | | 2.42±0.16 | | 6.5±0.22 | 75.2±13.7 |

**Table S5. Macrocopic mechanical date for uniaxial compression at room temperature**

|  | YBCO-3D | EP | EPIP | TSMTG |
| --- | --- | --- | --- | --- |
| **Yield Strength**  **(MPa)** | 5.34±0.54 | 135.47±5.59 | 151.50 ±3.50 | 200.37±16.78 |
| **Static Toughness**  **(KN m-2)** | 620±140 | 47210±1750 | 48740±6170 | 1360±320 |
| **Yield Strain**  **(%)** | 11.01±0.30 | 10.01±1.21 | 6.76±1.10 | 1.37±0.16 |
| **Elastic Modulus**  **(GPa)** | 0.065±0.010 | 1.97±0.28 | 3.12±0.58 | 20.63±1.62 |

**References**

[1] B. Zhang, Q. Zhang, P He, Y. Ma, L. Shen, X. Zhang, Y. Zhou, *Adv. Funct. Mater.* 2021, 31, 2100680.

[2] A. I. Kuzmenkov, A. M. Tesker, P. E. Kazin, Y. D. Tretyakov, *Phase Transitions* 1993, *41*, 165.

[3] C. Legros, C. Haut, L. Ponsonnet-Mora, J. Ayache, *J. Eur. Ceram. Soc.* 1999, *19*, 165.

[4] N.R. G. Kaiser, H. R. Khan, *Cryogenics* 1993, *33*, 124.

[5] S. Jin, T. H. Tiefel, R. C. Sherwood, M. E. Davis, R. B. van Dover, G. W. Kammlott, R. A. Fastnacht, H. D. Keith, *Appl. Phys. Lett.* 1988, *52*, 2074.

[6] Jin, S, Tiefel, T H, Sherwood, R C, van Dover, R B, Davis, M E, Kammlott, G W, & Fastnacht, R A. *Phys. Rev.* B. 1988, 37, 13

[7] D. K. Namburi, Y. Shi, K. G. Palmer, A. R. Dennis, J. H. Durrell, D. A. Cardwell, *J. Eur. Ceram. Soc.* 2016, *36*, 615.

[8] Y. Setoyama, J. Shimoyama, T. Motoki, K. Kishio, S. Awaji, K. Kon, N. Ichikawa, S. Inamori, K. Naito, *Physica C: Superconductivity and its Applications* 2016, *531*, 79.
